# Interleukin-27 levels in patients with myasthenia gravis

**DOI:** 10.1515/tnsci-2020-0134

**Published:** 2020-09-09

**Authors:** Xiao-Jiao Liu, Lin-Jie Zhang, Ming Yi, Li-Min Li, Jing Wang, Yuan Qi, Peng Zhao, Da-Qi Zhang, Li Yang

**Affiliations:** Department of Neurology, Tianjin Medical University General Hospital Airport Hospital, Tianjin, China; Department of Neurology, First Affiliated Hospital of Hainan Medical University, Haikou, China

**Keywords:** interleukin-27, myasthenia gravis, quantitative MG score, thymoma-associated myasthenia gravis, MG activities of daily living

## Abstract

Interleukin-27 (IL-27), which belongs to IL-12 family, influences the function of T cells (Tregs) through regulating the expression, and function of forkhead box P3 (FoxP3). In this study, we detected the IL-27 serum levels in 59 myasthenia gravis (MG) patients and 35 healthy controls (HCs). Among them, 32 MG patients received immunoglobulin intravenous (IVIG) injections (0.4 g/kg per day for 5 consecutive days). IL-27 levels were collected before and after the treatments and subjected to a comparative study. Finally, we assessed the correlations of IL-27 levels with the clinical characteristics of MG. As a result, serum IL-27 levels were significantly higher in MG patients than those in the HCs. Meanwhile, significant reduction was detected after the IVIG treatment. IL-27 levels positively correlated with both MG activities of daily living and quantitative MG score. IL-27 may participate in the pathogenesis of MG and can be used as an early marker for the diagnosis and prognosis of MG. In addition, IL-27 can be used as a target for MG treatment through the regulation of specific immune signaling and maintaining immune homeostasis.

## 1 Introduction

T-cell-mediated immune inflammation plays an important role in the pathogenesis of myasthenia gravis (MG). The differentiation imbalance in the cluster of four positive T-helper cells (T helper, Th) leads to an increase in Th1 and Th17 numbers and abnormal cytokine production, including interleukin-1 (IL-1), IL-6, IL-17, interferon-γ, and tumor growth factor α. This causes an imbalance in immune homeostasis and subsequently activates effector B cells that produce autoantibodies. Regulatory T cells (Tregs) play a key role in maintaining immune tolerance. Abnormal functions of Tregs have been reported in many autoimmune diseases including MG, multiple sclerosis (MS), and inflammatory bowel disease (IBD) [1,2]. Tregs are mainly divided into natural Tregs which are produced by thymus and express the transcription factor forkhead box P3 (FoxP3), induced Tregs, which are generated *in vitro*, and IL-10-secreting type 1 regulatory T cells (Tr1) according to the differences in cell surface antigens, cytokine produced, and mechanism of actions [3,4]. FoxP3 is a key transcription factor that determines the development of Tregs and its expression of immunosuppressive molecules, which is an essential element for Tregs to exert their regulatory functions [4]. Tregs maintain immune homeostasis through various ways including secretion of anti-inflammatory factors including IL-10, IL-35, and transforming growth factor-β; adhesion with cell surface molecules such as CD25 and cytotoxic T-lymphocyte antigen 4; and secretion of perforin or granzyme, which directly inhibits or kills effector cells such as antigen presenting cells (APCs) and B cells that produce autoantibodies [5,6].

IL-27 is a cytokine that belongs to the IL-12 family, which is mainly secreted by activated APCs, and is composed of a heterodimeric structure containing Epstein–Barr virus-induced gene protein 3 and p28 subunits. IL-27 plays an immunosuppressive role by inducing the expression of transcription factors c-Maf, AhR, Egr-2, and Blimp-1 through STAT1 and STAT3 signaling pathways, promoting the differentiation of Tr1 and the production of IL-10, which subsequently promotes the further production of IL-10 by Tr1, and eliminating the self-reactive B cells [7,8]. Cox et al. found that IL-27 restricts their conversion by limiting the model of colitis T-cell transfer and ovalbumin-dependent tolerance [9], resulting in a decreased number of Tregs. IL-27 neutralization (by p28-specific antibody) reduced the severity of graft-versus-host disease [10]. Belle et al. found reconstitution of Treg, increased FoxP3 expression, and promoted expression of IL-10 by Tregs in the IL-27 receptor defect mice [10]. However, Kim et al. demonstrated that while CD4^+^CD25^+^ T-cell transplantation was able to induce colitis in the recipient mice, CD4^+^CD25^+^ T cells from IL-27 receptor defect donor mice failed to do so due to a defect in T-cell survival [11]. The regulatory effect of IL-27 on Treg has been widely accepted with the changes in different diseases and animal models. However, only few studies have investigated the functions of IL-27, IL-27 receptor subunit alpha, and FoxP3 in MG. In this study, we detected the serum IL-27 levels of patients with MG and discussed its association with the severity and prognosis of the disease.

## 2 Materials and methods

### 2.1 Subjects

The study was a prospective study and mainly described 59 patients with MG and 35 healthy controls (HCs) who were all enrolled in Tianjin Medical University General Hospital from January 2015 to January 2018. Patients with MG were subcategorized according to their disease subtypes, which included 20 cases of ocular MG (OMG), 39 cases of generalized myasthenia gravis (GMG), 15 cases of thymoma-associated MG (TAMG), and 49 cases of anti-acetylcholine receptor (AChR)-positive MG. All patients did not receive any cholinesterase inhibitor or immunosuppressive agent treatment 6 months prior to this study. The diagnostic criteria of MG were based on an experienced neurologist. The clinical diagnosis of MG was mainly based on medical histories and clinical manifestations (fluctuating skeletal muscle weakness and fatigue) of the patients, and at least one positive result from the following indicators: antibody levels (AChR and muscle-specific tyrosine kinase [MuSK] antibody), electrophysiological results (repeated nerve stimulation or single-fiber electromyography), and response to the therapeutic effects of acetylcholinesterase inhibitor. Patients in this study did not suffer from any migraine, muscular dystrophy, paraneoplastic neurological syndromes, hyperthyroidism, or any other systemic diseases which can also cause muscle weakness. The exclusion criteria included infection, autoimmune diseases, and cancers. Patients with one of the following conditions were also excluded: allergic to immunoglobulin, serum IgA levels are lower than 5% of the normal minimum limit, severe heart disease, renal insufficiency, and thrombotic events.


**Informed consent:** All patients have signed informed consent forms.
**Ethical approval:** The research is in compliance with all the relevant national regulations, institutional policies, and in accordance with the tenets of the Helsinki Declaration. This study was approved by the ethics committee of Tianjin Medical University General Hospital (Ethical No. IRB2019-WZ-131).

### 2.2 Clinical evaluation and sample collection

Demographic features, MG Foundation of America (MGFA) classification, quantitative MG (QMG), MG-activities of daily living (MG-ADLs) score, thymus CT, electrophysiology test, and therapy data were obtained from the MG database. Anti-AChR antibody and anti-MuSK antibody were detected by cell-based immunofluorescence assay. Plasmids of AChR and MuSK were donated by Professor Angela Vincent and Professor David Beeson (University of Oxford). Patients were clinically assessed according to their scores in MG-ADLs and QMG and postintervention status of MG defined in accordance with MGFA. Serum samples of 59 patients were collected before immunoglobulin intravenous injection (IVIG) treatment, followed by 32 matched samples collected again at minimal manifestation-3 status after IVIG treatment.

### 2.3 ELISA-based measurement of serum IL-27 levels

Serum IL-27 levels were detected using a human IL-27 enzyme-linked immunosorbent assay (ELISA) kit (e-Bioscience, San Diego, CA, USA), according to the manufacturer’s protocol. Generally, serum samples and standard solutions were incubated with antibody-coated plates according to the manufacturer’s protocol. Subsequently, 50 µL of diluted biotin-conjugate, 100 µL of diluted streptavidin–HRP, and 100 µL of TMB substrate solution were subsequently incubated at room temperature for 2 h, 1 h, and 30 min, respectively. Stop solution of 100 µL was used to stop the reaction. The sample was directly used for detection without dilution and was repeated three times to obtain the average value. The optical density was read at 450 nm using a microplate reader (SYNERGY 2; BioTek, USA). Serum IL-27 levels were determined according to the standard curves. The minimum threshold concentration of IL-27 was 9.5 pg/mL.

### 2.4 Statistical analysis

Statistical analysis was carried our employing Statistical Package for the Social Sciences 22.0 (SPSS Inc., Chicago, IL, USA). GraphPad PRISM Version5 (Graph Pad Software Inc., San Diego, CA, USA) was used to generate graphs. The serum IL-27 levels between different MG subgroups and HCs were compared by analysis of variance. A paired *t* test was used to compare serum IL-27 levels before and after treatment. Spearman or Pearson was used for correlation analysis between IL-27 and scores of QMG or MG-ADLs. Differences or correlations with *P* values of <0.05 were considered as statistically significant.

## 3 Results

### 3.1 Demographic and clinical features of subjects

Demographic and clinical features of the subjects are displayed in Table 1. A total of 35 HCs (17 females and 18 males) without any acute or chronic inflammatory diseases were recruited at the Medical Examination Center of Tianjin Medical University General Hospital. All MG patients included in this study were diagnosed and hospitalized for the first time with exceptions for OMG patients, which did not progress to GMG during the first 2 years of follow-up. The OMG patients were recruited at the outpatient clinic. They have not received any treatment (poor acetylcholinesterase inhibitor effects or side effects of immunosuppressive agents) within the past 6 months and have been followed up. All enrolled patients underwent MGFA clinical classification to assess their severity of the disease. Most of them were classified as MGFA Classes I–III, except for nine patients who were classified as MGFA V. During second month of follow-up, two patients died from respiratory failure. From a total of 59 patients, 32 patients completed enrollment before and after IVIG treatment (20 OMG, 2 deaths, and 5 transferred patients). Most patients were positive for anti-AChR antibody and 10 patients were seronegative, while all patients were negative for anti-MuSK antibody. This study did not detect LRP4 antibody.

**Table 1:** Demographic and clinical features of MG and HCs

Characteristics	Patients with MG (*n* = 59)	Controls (*n* = 35)	*P* value
Age at disease onset (years)	54.97 ± 14.54	55.31 ± 11.02	0.81
Female:male	20:39	17:18	0.64
MG-ADLs	5.90 ± 4.12	—	—
MG-QMG	10.25 ± 6.06	—	—
MGFA	I–V	—	—
GMG:OMG	21:10	—	—
Thymoma	15/59(25.42%)	—	—
AChR-Ab (+)	49/59(83.05%)	—	—

GMG, generalized myasthenia gravis; OMG, ocular myasthenia gravis; MG-ADLs, MG activities of daily living; MGFA, MG Foundation of America classification; QMG, quantitative MG score; AChR-Ab (+), anti-AChR antibody positive.

### 3.2 Serum IL-27 levels increased before treatment and decreased after treatment and positively correlated with QMG and MG-ADLs

**Figure 1:**
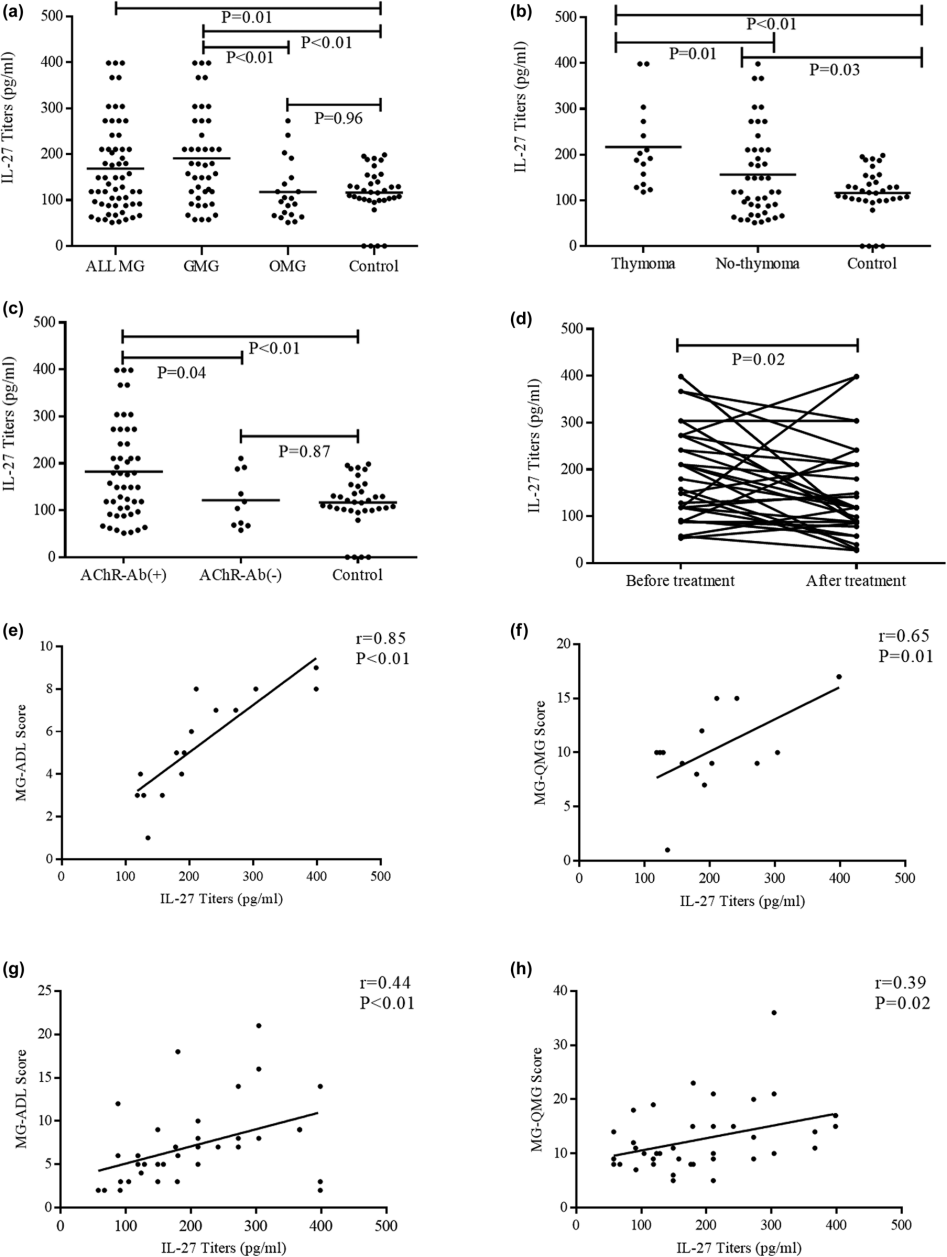
Serum levels of IL-27 in different subtypes of MG and HC and correlated with QMG and ADL in patients with MG. (a) Serum IL-27 titers in 59 patients with MG, including 39 GMG, 20 OMG, and 35 HCs. (b) Serum IL-27 titers in MG patients with or without thymoma. (c) Serum IL-27 titers in MG patients with or without AChR antibody. (d) Serum IL-27 titers in MG patients before and after treatments. The relationship between the serum IL-27 titers with MG-ADLs (e) and QMG score (f) in MG patients with thymoma. The relationship between the serum IL-27 titers with MG-ADLs (g) and QMG scores (h) in GMG.

Serum IL-27 levels in patients with MG (168.44 ± 97.98) are higher than those in HCs (116.60 ± 53.13). Serum IL-27 levels varied in different MG subtypes. The IL-27 levels in MG patients in GMG subtype (190.93 ± 101.07) were significantly higher than those in OMG (117.69 ± 63.82, *p* < 0.01) and HCs (116.60 ± 53.13, *p* < 0.01; Figure 1a). Patients with TAMG (216.75 ± 91.31) had significantly higher IL-27 levels than both no-TAMG (NTAMG, 156.51 ± 94.92, *p* = 0.01; Figure 1b) and HCs (116.60 ± 53.13, *p* < 0.01; Figure 1b). The serum IL-27 levels of AChR-MG patients (182.13 ± 100.43) were significantly higher compared with AChR(−)-MG patients (121.36 ± 57.67, *p* < 0.01) and HCs (116.60 ± 53.13, *p* < 0.01; Figure 1c). Again, serum IL-27 levels in patients with OMG (117.69 ± 63.82) and AChR(−)-MG (121.36 ± 57.67) were not statistically significant than HCs (116.60 ± 53.13) but directionally consistent (*p* = 0.96, Figure 1a; *p* = 0.87, Figure 1c). Meanwhile, NTAMG patients had significantly higher serum IL-27 levels than HCs (116.60 ± 53.13, *p* = 0.03; Figure 1b). The serum IL-27 levels significantly decreased after IVIG treatments, as evidenced by the comparative study conducted in the 32 patients (before the treatment: 198.34 ± 104.28 and after the treatment: 144.14 ± 100.35, *p* = 0.02; Figure 1d).

Positive correlations between serum IL-27 levels and scores of MG-ADLs or QMG were identified in TAMG and GMG groups, respectively (*r* = 0.85, *p* < 0.01; *r* = 0.65, *p* = 0.01, Figure 1e and f; *r* = 0.39, *p* = 0.02; *r* = 0.44, *p* < 0.01; Figure 1g and h). Meanwhile, no statistically significant correlations were observed between the serum IL-27 levels and scores of MG-ADLs or QMG in either of the subgroups of anti-AChR antibody-positive or -negative MG (*r* = 0.24, *p* = 0.09; *r* = 0.23, *p* = 0.11; *r* = 0.31, *p* = 0.39; *r* = 0.35, *p* = 0.33); in addition, no significant correlation was found between serum IL-27 levels and OMG (*r* = 0.59, *p* = 0.11; *r* = 0.43, *p* = 0.06).

## 4 Discussion

Previous studies have demonstrated the association of rheumatoid arthritis with the increase in IL-27 levels in serum, synovial membranes, and synovial fluid. The serum IL-27 levels were correlated with disease activity [12,13]. Similar results were observed in patients with psoriasis [14]. Serum IL-27 decreased during the acute phase of the disease and increased after immunomodulatory therapy in MS [15]. Another study found that IL-27 levels were increased in cerebrospinal fluid and active plaque in MS [16]. IL-27 administration in experimental autoimmune meningococcal meningitis mice attenuated the degree of disease and disability [17]. Meanwhile, the same intervention constructs in Tregs IL27Ra knockout mice cannot effectively relieve the clinical symptoms [17]. Besides, serum IL-27 levels were elevated in patients with IBD during the acute phase of the disease [18]. IL-27 stimulation enhanced Tregs functions and FoxP3 expression *in vivo* [18]. While the IL27Ra-defected Tregs cannot exert inhibitory functions *in vitro* [18]. IL-27 levels can be either increased or decreased in MG patients, based on their disease stages and treatment status. Previous study found that the serum IL-27 levels were higher in MG patients than those in HCs [19], which was consistent with our results.

Previous studies demonstrated the functions and number of Tregs and the expression of FoxP3 reduced in TAMG [20,21]. Meanwhile, other studies observed that the reduction in Tregs inhibitory function leads to increased production of autoantibody, which intensified the severity and contributed to the progression of AChR-MG [22,23,24]. Jeong and his colleagues demonstrated that higher IL-27 levels were observed in early-onset myasthenia gravis (EOMG) and the TAMG subpopulation contains lower IL-27 levels [19]. In our study, IL-27 levels in TAMG and AChR-MG were higher than those in NTAMG and AChR(−)-MG, respectively. Most importantly, IL-27 levels were positively correlated with both QMG and MG-ADLs. We speculated that the IL-27 may affect the function or quantity of Tregs. However, the exact relationship between IL-27 levels, Treg function, and FoxP3 expression in MG was not clear; therefore, further studies were needed. IL-27 promoted the production of IL-21, which promoted the transformation of anti-AchR antibody and in worse case the pathological process of MG [25]. Besides, their subsequent research showed that the serum IL-27 levels in the anti-AchR antibody-positive group were positively correlated with the antibody concentrations [26]. About 80–85% of patients in GMG were positive for anti-AChR antibodies, which was higher than that in OMG [27]. These findings combined with Jeong et al. study explain why patients with GMG have higher levels of IL-27 than those with OMG. In recent years, the effects of IVIG treatments on Tregs functions have been studied in different autoimmune diseases [28,29,30,31]. IVIG treatments affected the Treg functions and the regulation of inflammatory factors through altered APC functions [32,33], while IL-27 was mainly secreted by activated APCs [34]. Our study found that the serum IL-27 levels in patients with MG significantly decreased after the IVIG treatments. However, the underlying mechanisms and signaling pathways involved in this process were still not clear.

IL-27 exerts different immunomodulatory effects in different diseases, even in different periods of the same disease. The main factors are related to its potential effector pathway, disease period, and the presence or absence of interacting regulatory cytokines or T-cell subsets [35]. Both increase and decrease in IL-27 levels may only be a manifestation. Further studies of the underlying mechanisms are needed for the clinical transformation and application of targeting IL-27 in the treatments of MG. Here, we proposed a scientific hypothesis that IL-27 may participate in the pathogenesis of MG by acting on Tregs and may serve as an early marker for the diagnosis and prognosis of MG. Most importantly, IL-27 can be used as a new target for the clinical treatments of MG through regulating specific immune signaling and maintaining immune homeostasis. Therefore, further study is worth in order to clarify the role of IL-27, IL-27Rα, and FoxP3 in the progression of MG, and to study the association between IL-27 levels and the clinical characteristics of MG.

## 5 Conclusions

Taken together, our study demonstrated that serum IL-27 levels were significantly increased in patients with MG and decreased after IVIG treatments. Positive correlations were identified between serum IL-27 levels and scores of MG-ADLs and QMG, although the increase or decrease in IL-27 levels may only be a manifestation. Further studies of the underlying mechanisms are needed for the clinical transformation and application of targeting IL-27 in the treatment of MG.
